# Effects of Particle Size Distribution of Standard Sands on the Physical-Mechanical Properties of Mortars

**DOI:** 10.3390/ma16020844

**Published:** 2023-01-15

**Authors:** Ruan L. S. Ferreira, Mauricéia Medeiros, Jéssyca E. S. Pereira, Glauco F. Henriques, Jennef C. Tavares, Markssuel T. Marvila, Afonso R. G. de Azevedo

**Affiliations:** 1 Civil Construction Department, Federal Institute of Education, Science and Technology of Pernambuco, Pesqueira 55200-000, Brazil; 2 Chemical Engineering Department, Federal University of the Rio Grande of the Norte, Natal 59078-970, Brazil; 3 Graduate Program in Civil and Environmental Engineering-PPGECAM, Federal University of Paraíba, Campus I Lot. Cidade Universitaria, João Pessoa 58051-900, Brazil; 4 Science and Technology Department, Federal Rural University of Semi-Arid, Caraúbas 59780-000, Brazil; 5 Campus Rio Paranaiba, Federal University of Viçosa, Rio Paranaiba 38810-000, Brazil; 6 LECIV—Civil Engineering Laboratory, UENF—State University of the Northern Rio de Janeiro, Campos dos Goytacazes, Rio de Janeiro 28013-602, Brazil

**Keywords:** cementitious materials, particle size, granular skeleton, fine aggregate

## Abstract

Obtained natural sands can present different particle size distributions (PSD), although they have the same mineralogical origin. These differences directly influence the physical and mechanical behavior of mortars and, therefore, the performance of mortar and ceramic renderings. Standardizing the particle size of sands based on pre-established requirements in normative standards (NBR 7214 or ASTM C778) is one way to minimize these effects. However, these standards do not consider the optimization of the granular skeleton through the analysis of bulk density and PSD, which may be insufficient to obtain satisfactory results. Therefore, this paper analyzes the effects of using different particle size ranges on the physical and mechanical behavior of cement and hydrated lime mortars. The properties of consistency index, bulk density, air content, capillary water absorption, water absorption by immersion, flexural strength, compressive strength, and dynamic modulus of elasticity were evaluated. For this purpose, standardized sands of the same mineralogical origin were made with different particle size ranges, being: (i) standardized sand constituted by 25% of coarse and fine fractions (S25-control), (ii) standardized sand constituted by 30% of coarse fraction and 20% of fine fraction (S30-20), and (iii) standardized sand composed by 40% of coarse fraction, and 10% of fine fraction (S40-10), respectively. The results indicated that variations in the particle size composition of the standardized sands are necessary to obtain mixtures with higher compactness and, therefore, mortars with better physical and mechanical performance. Thus, the dosage of the particle size fractions of standardized sand should consider the optimization of the granular skeleton, being the unit mass and the granulometric composition as important parameters to meet this premise.

## 1. Introduction

The consumption of natural resources and energy worldwide has increased considerably due to urban and economic growth. It is estimated that the construction industry consumes 40% of these natural resources [1], and this demand is expected to double by 2050 [2]. As a result, this industry is considered one of the sectors that contributes the most to changing the environment.

For example, Brazil is considered one of the countries that extract and consume the most natural resources in an uncontrolled, non-optimized way. Aggregates, for example, are materials with high consumption in the country. In 2019 only, about 2.5 tons of aggregates were consumed per inhabitant [3], which corresponds to a consumption of approximately 523 million tons. In particular, regarding the sand used in construction (natural and artificial), data from the Departamento Nacional de Produção Mineral-DNPM [4] pointed out that in 2015 about 349 million tons of sand were consumed. These data highlight the importance of the rational use of these natural resources for the preservation of the environment. However, this is not an isolated problem in Brazil and happens in other countries around the world. Li et al. [5] and Zhang et al. [6] highlight the great urgency for alternative sources of aggregates in China, due to the high production of concrete. The same is highlighted by other authors in other regions of the world, like the USA [7] and Europe [8].

Among the various materials used in the construction industry, mortar is one of the main materials used due to its mechanical properties, durability, cost-effectiveness, and availability [9]. Cementitious mortar can be described as a combination of fine aggregates (typically riverbed sand), hardened cement paste, and interfacial transition zones. The physical-mechanical behavior of mortars is highly related to the properties of the aggregate used due to its large volume fraction in the mixture [10].

Some of the properties of aggregates such as textural parameters of aggregate grains, such as grain size (expressed in terms of grain size composition), shape, surface texture, and mineralogical composition significantly affect the behavior of construction materials [11,12,13,14,15,16]. In addition, other aggregate factors related to the characteristics of the particle system, the number of grains in each fraction, and how these particles are organized in their equilibrium position should be considered [17].

Notwithstanding, currently one seeks to produce construction materials with less cement and simultaneously present adequate mechanical and physical performance. In this context, optimizing the properties of a mortar mixture considering the maximum packing density of the constituent materials [18] is an important alternative in the production of efficient materials.

The shape and particle size distribution of the aggregates are parameters that are related to obtaining the maximum packing density. Such influence is of great interest since the internal microstructure of cement-based mortar is a function of its porosity and pore size distribution (the result of voids between sand grains and those intrinsically present in cementitious products) [19]. Thus, a change in sand grain size (the main component by weight and volume) should have a drastic effect on mortar microstructure and hence on mechanical properties, porosity, hydration of materials, as well as other characteristics [20,21,22].

In this perspective, the Brazilian (NBR 7214) and American (ASTM C778) standards determine that natural or artificial sands should be standardized in terms of particle size fraction before being used in tests for determining the strength class of cements or evaluation of the pozzolanic activity of supplementary cementitious materials (SCM). For example, Brazilian standards recommend that standardized sand should have four size fractions: fraction #16 with particle size between 2.4 and 1.2 mm, fraction #30 with particle size between 1.2 and 0.6 mm, fraction #50 with particle size between 0.6 and 0.3 mm, and fraction #100 with particle size between 0.3 and 0.15 mm. However, these standards do not take into account the effects of the particle size distribution (PSD) on mortar behavior, which can be a determining factor for the acceptance or not of a particular cement or SCM.

Thus, the effects of using standardized natural sands (SS) with different size fractions on the physical and mechanical properties of cement and lime mortars were investigated. The results obtained show that the production of SS based on the maximum packing density concept is extremely necessary for the rational use of natural aggregates and for obtaining mortars with better physical and mechanical properties. This initiative can, in the long run, contribute to mitigating environmental impacts and avoiding waste of one of the main materials in the construction industry.

**Figure 1:**
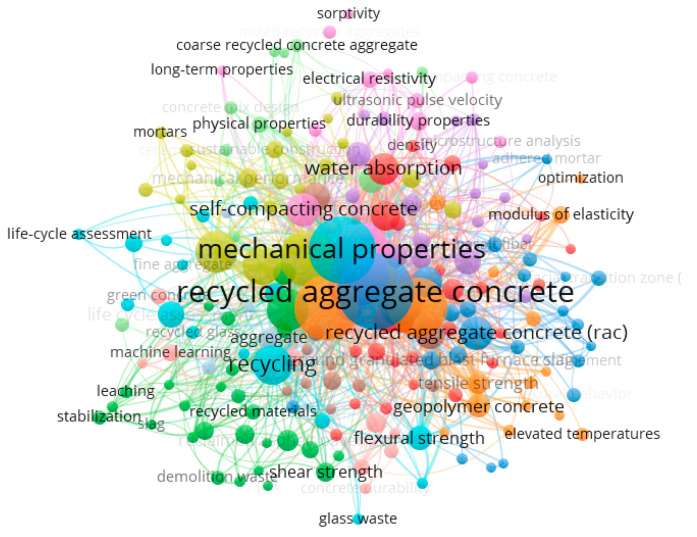
Bibliometrics analysis from [23].

The main novelties of the research are related to the development of an experimental program that looks for the effect of the granulometry of the sands in the main mortar properties. Marvila et al. [23] carried out bibliographical research on aggregates in mortar, developing a bibliometric study highlighted in Figure 1. The results obtained were extracted from the Scopus database based on articles published between 2013 and 2022, using the keywords recycled; aggregate; concrete (or mortar); and construction. Note the absence of words such as granulometry, packaging, and relative density, although the term self-compacting concrete appears. This illustrates the need for research that evaluates experimental results on the influence of standardized sand granulometry, especially in mortars, since this is one of the main parameters of aggregates such as sand.

## 2. Experimental Program

### 2.1. Materials

Pozzolanic Portland cement (PC) (similar to ASTM C 595 Portland Pozzolanic) was used, with a specific gravity of 2.93 g/cm^3^, a specific surface BET of 4.362 m^2^/g, Blaine fineness of 4000 cm^2^/g, and compressive strength of 32.7 MPa at 28 days. The cement used has approximately 20% of pozzolanic material and the rest of clinker and was chosen due to its high use in the region where the research was carried out. As the complementary binder, hydrated-lime (HL) type CH-I (similar to ASTM C207-6) with a specific gravity of 2.30 g/cm^3^, bulk density of 0.56 g/cm^3^ and CaO and MgO oxide contents higher than 95% were used. The use of HL is justified by its beneficial effect on mortar properties (such as workability, water retention, and ability to absorb deformations) and by reducing cement consumption [24,25,26,27], contributing to minimizing environmental impacts from clinker production.

As fine aggregate was used, quartz sand obtained from the riverbed with a specific gravity of 2.62 g/cm^3^. After collection, this sand was dried in an oven (105 ± 5) °C for 48 h and then sieved to remove particles with size >4.75 mm. After this procedure, three types of standardized natural sands (SS) with different particle size distributions (PSD) were made, based on the Brazilian standardization NBR 7214 [28]. Table 1 shows the particle size fractions and percentages used to produce the three types of SS studied.

Table 2 presents the physical properties of the studied sands. By increasing the number of particles with larger particle size fractions, the fineness modulus increases and the content of fines (<0.15 mm) decreases. In Brazil there are no specific standards for aggregates used in mortars, however, the ASTM C144-3 [29] that specifies the aggregates used in mortars, limits the content of fine particles that pass the 0.15 mm mesh to 15%. Thus, all the sands studied meet the limit of fine particles established by the American standard. On the other hand, the sand with 30% coarse particles (SS30-20) has a higher unit mass and higher uniformity coefficient, which is appreciable because higher unit masses and uniformity coefficients favor the reduction of voids in the mixture and, consequently, contribute to improving the performance of mortars in the fresh and hardened states.

It should be noted that the SS-25 composition was chosen because it is the standard used in different international standards, as previously highlighted. Therefore, it is necessary to highlight the criteria for choosing SS30-20 and SS40-10 compositions: (i) set the fine particle limit established by ASTM C144-3 [29]; and (ii) increase the uniformity coefficient of the sands, favoring the reduction of voids. This analysis of the influence of the sand uniformity coefficient on the mortar properties was studied by Azevedo et al. [30]. This study was used as a basis for defining the SS30-20 and SS40-10 compositions.

Another important piece of information is to note that Table 2 shows similar properties for SS-25, SS30-20, and SS40-10 sands. This is fundamental because the objective of the article is to evaluate the influence of the material’s granulometry on the properties of the mortar. Therefore, the other properties established in Table 2 need to be similar, except for the uniformity coefficient, which is impossible to standardize with a change in granulometry.

**Table 2:** Physical properties of sands.

Physical Properties	Standardization	SS-25	SS30-20	SS40-10
Maximum size (mm)	NBR NM 248 [31]	2.36	2.36	2.36
Fineness modulus	NBR NM 248 [31]	2.353	2.535	2.965
Fines content (%) ^1^	-	2.99	2.77	1.84
Bulk density (kg/m^3^)	NBR NM 45 [32]	1.634	1.660	1.633
Uniformity coefficient	-	4.10	4.70	4.40

^1^ Determined by the amount of material that passes the sieve with an aperture of 0.15 mm.

**Figure 2:**
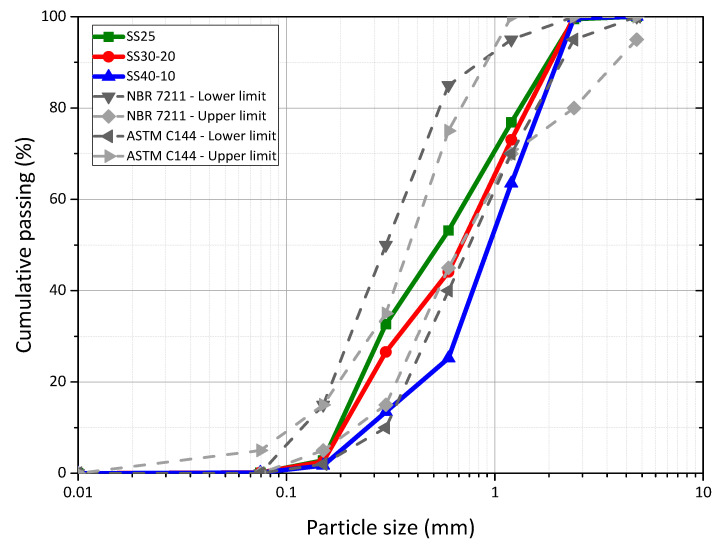
Sieve size curve of the sands studied and the respective normative limits.

Figure 2 shows the particle size distribution of the studied sands, determined according to the Brazilian standard NBR NM 248 [31]. Regarding the standard limits, SS25 and SS30-20 are close to the ranges recommended by ASTM C144-03 [29] and NBR 7211 [33].

SS40-10 sand is outside the normative ranges established in American and Brazilian standards and, therefore, does not meet the requirements for natural fine aggregates used in mortars and concretes, respectively. These results show that the predominance of fractions with larger sizes worsens the particle size distribution of the sands. However, a limit of 30% of “coarse” particles and 20% of “fine” particles improves the granular skeleton, justifying the higher uniformity coefficient and higher bulk density of composition SS30-20. The relationship between the bulk density and the particle size distribution of the fine aggregates in particle packing was also investigated in a previous study [34] and is shown to be important to predict the mechanical behavior of mortars [35].

### 2.2. Methods

The following criteria were established to produce the mortars:

- Maintain the mixing proportion of 1:1:6 by weight of Portland cement, hydrated lime, and sand for all mixtures, respectively. The choice of this mixture proportion was due to its wide use in civil construction works in the region and because it is a mixing ratio used in previous research [25,26,27];
- Mortars are mixed in a mortar, according to NBR 16541 [36]
- The production of three types of mortar: MSS25 (reference), MSS30-20 and MSS40-10;
- The consistency index of the mortars was kept constant at 260 mm in all mixtures according to NBR 16541 [36];
- The ideal amount of water for mortar mixture was determined experimentally to ensure workability. The procedures adopted followed the specifications of NBR 13,276 [37]

Table 3 shows the amount of each material used in the mortar production. For laboratory mixing, using a planetary mechanical mixer with a capacity of five and the procedures followed the specifications of NBR 16,541 [36].

The increase in the number of coarse particles increased the water consumption for the mortars to reach the consistency of 260 mm (Table 2), which consequently increases the water/binder ratio (w/b) of the mixtures produced with SS40-10. Although it seems to be contradictory because the amount of fine particles decreases (i.e., the specific surface to be wetted also decreases), this behavior can be attributed to the packing effect of the granular skeleton. In this sense, the standardized sands that have lower bulk density and worse PSD (SS25 and SS40-10) have more voids and, therefore, require a greater amount of water to meet the established flow requirements (260 mm). These results corroborate those obtained in previous studies [13,22,34,38,39]

The tests performed for the characterization of the mortars in the fresh and hardened states are listed in Table 4. For the characterization of mortars in the hardened state, prismatic specimens with dimensions of 40 mm × 40 mm × 160 mm were used.

The molding and compaction of the specimens followed the recommendations of NBR 13279 [46]. During the first 48 h, the surface of the specimens was protected with a glass plate to avoid evaporation of the mixing water. After this period, demolding was performed, keeping the specimens exposed to air and at room temperature (25 ± 5 °C) for 28 days for each test, as shown in Table 3. 

The mortars’ dynamic modulus of elasticity was evaluated using ultrasonic dynamic technique and non-destructive tests. The dry density of the hardened mortar (as per NBR 13280 [47]-equivalent to EN 1015–10) and the ultrasonic speed of the specimens (as per NBR 8802 [48]-equivalent to ASTM C 597–16) were determined for this purpose. For the various types of mortars, the Poisson coefficient was constant and equal to 0.20.

The test of weight variation of the mortars over time was based on the weighing in precision analytical balance of prismatic specimens (40 mm × 40 mm × 160 mm) during a period of 28 days counted from the demolding, according to the specific Modalités D’essais of the CSTB [43]. This test makes it possible to evaluate the behavior of mortars regarding weight loss and gain during their hardening [19] and, consequently, allows the observation of the evolution of mortar hardening resulting from cement hydration reactions, and the carbonation reaction of calcium hydroxide by the action of carbon dioxide [39]. From the weights obtained, the weight variation rate (*dm*/*dt*) was determined based on Equation (1).

(1) $$dm/dt=\frac{m_{0}-m_{n}}{m_{b}}$$

where, $m_{0}$ is the initial weight of the specimen on the day of demolding (48 h after molding) and $m_{n}$ and $m_{b}$ correspond to the weight of the specimen after (1, 2, 3...*n*) days and the weight of the anhydrous binder in the mortar, respectively.

## 3. Results and Discussion

### 3.1. Properties of Fresh Mortar

#### 3.1.1. Bulk Density

**Figure 3:**
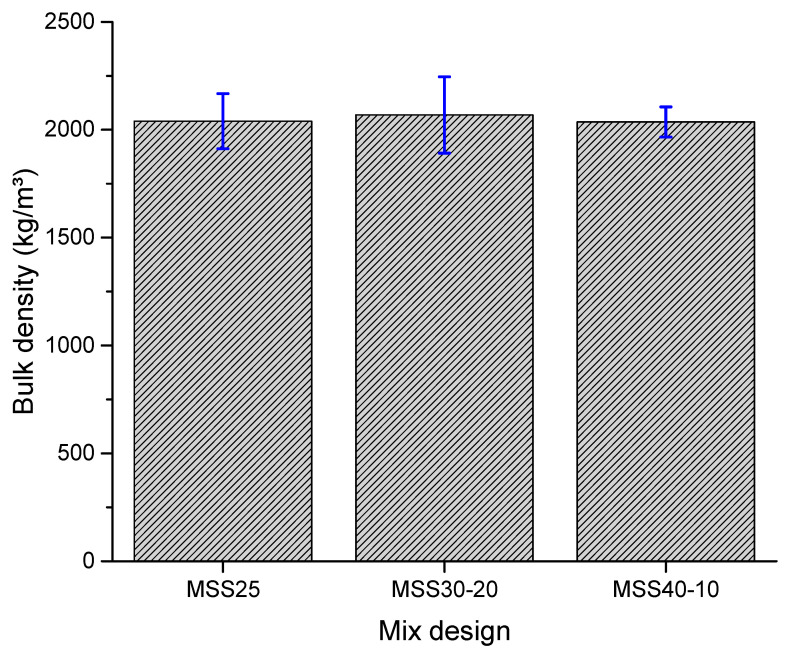
Bulk density of fresh mortars.

Figure 3 shows the results of the bulk density tests of the mortars in the fresh state. The mortars presented similar bulk density due to the specific masses of their constituents being equal. However, a slight increase in bulk density was observed in the mortars produced with 30% coarse particles (MSS30-20). This is due to the better particle size distribution of the SS30-20 sand, which contributes to obtaining more compact mortars in the fresh state and, therefore, with higher bulk density. These results also show that there is a limit to the amount of coarse particles supported by the system for maximum mortar compaction, being ideal for this property the use of standardized sands with up to 30% of “coarse” fractions and 20% of “fine” fractions.

These results reveal that all mortars studied can be classified as normal [18], thus not limiting their use in coatings. In practical terms, using mortars with adequate density reflects a better workability, since it is possible to reduce the effort employed in its application and, consequently, increase productivity. Comparing the results with other studies that used a similar composition, it is observed that Marvila et al. [49] obtained bulk density values of 2030 kg/m^3^, while Ferreira et al. [27] obtained values of approximately 2040 kg/m^3^. Therefore, the results obtained are coherent.

#### 3.1.2. Air Content

**Figure 4:**
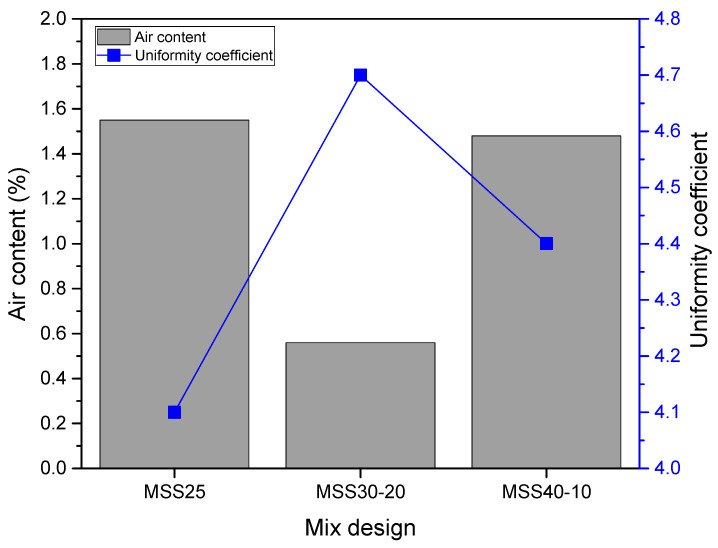
Air content of fresh mortars.

Figure 4 shows that the modification of the particle size composition of the SS25 standardized sand contributes to the reduction of the incorporated air content of the mortars when compared to the reference mortar (MSS25). This improvement was observed mainly when using standardized sand with 30% “coarse” and 20% “fine” fraction (SS30-20). The reason for the decrease in air content is the same as for the increase in fresh mass density. Thus, the use of standardized sands with better particle size distribution the reduction of voids, the number of macro-pores decreases, and, consequently, the volume of incorporated air also decreases. However, the mortar with sands composed of a larger amount of “coarse” particles (SS40-10) showed lower content of incorporated air when compared to the reference mortar, which contradicts the results of bulk density. In this case, the higher water demand of MPS40-10 contributed to this difference, probably due to the filling of voids by water.

It is also observed that by increasing the continuity of the granular skeleton of the sands, there is a decrease in the trapped air content of the mortars, confirming that there is an improvement in the behavior in terms of air incorporated into the mixture when the particle size distribution of the sands is optimized. These observations are consistent with a previous study [27].

It is also important to note that, generally, a higher content of air incorporated into fresh mortars can improve their workability [26], however, its excess is detrimental to mechanical strength [44,50], permeability, and adherence to the mortar substrate [51].

### 3.2. Properties of Hardened Mortar

#### 3.2.1. Capillary Water Absorption

**Figure 5:**
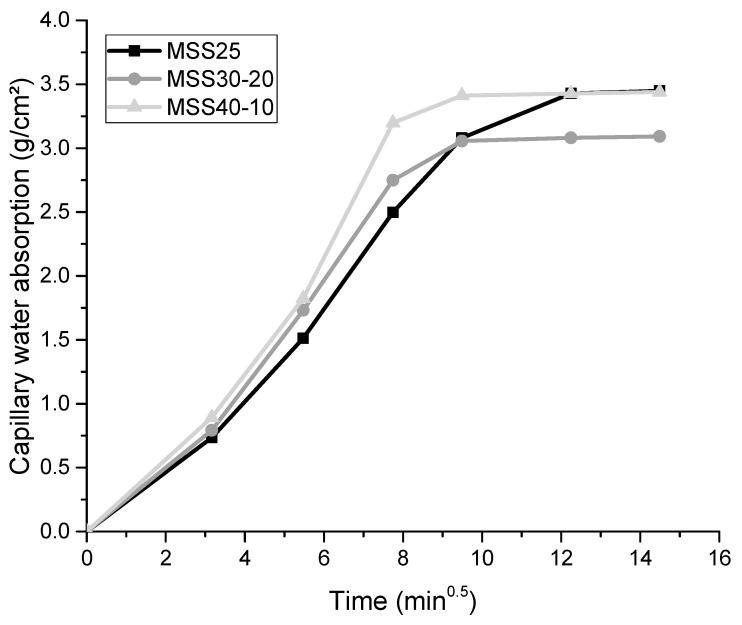
Capillary water absorption of hardened mortars.

Figure 5 shows capillary absorption curves as a function of time for the mortars studied. The results are consistent with the observations made in the anhydrous and fresh state. The mortars produced with SSS30-20 showed the best performance when compared to the other mortars. This behavior is justified by the optimization effect of the granular skeleton of the standardized sand with 30% coarse particles (SS30-20) and corroborates with the results obtained in the fresh state (i.e., higher bulk densities and lower incorporated air content). The worst performance obtained by the mortars MSS25 and especially by MSS40-10 can be attributed to the worst arrangement of the particles, reflecting the worst granulometric behavior of the aggregates and the lower fresh mass densities of the mortars. These results indicate that there is greater pore connectivity in the internal structure of the mortars produced with standardized sands SS25 and SS40-10.

Thus, in terms of capillary water absorption, the use of standardized sand with 30% of the “coarse” fraction and 20% of the “fine” fraction (SS30-20) results in mortars with reduced permeability, which hinders the entry of aggressive agents inside [26]. This behavior is important for the durability of coatings because, besides the ability to create compounds, water has the ability to degrade natural and artificial materials, becoming an essential factor behind most durability problems of cementitious matrices [16,52], especially in materials that are exposed to external environments. Comparing the results with other studies that used a similar composition, it is observed that the results are consistent with the research by Marvila et al. [53], who simulated the effect of porosity on capillarity properties. The results were around 3.5 g/cm^3^, similar to the present research.

#### 3.2.2. Water Absorption and Porosity

**Figure 6:**
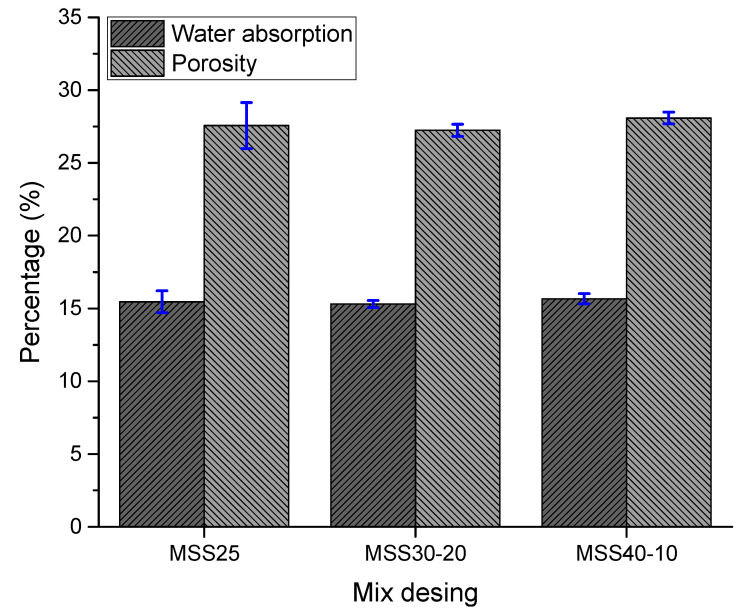
Water absorption and porosity of hardened mortars.

Figure 6 shows the results of the immersion water absorption tests in terms of water absorption and open porosity. No significant effects of using different particle size distributions of the standardized sand were observed on these properties. This behavior can be justified by the carbonation effect of lime [11,36] whose result is the filling and clogging of the surface pores and, consequently, a decrease in mortar porosity.

Mortars produced with SS25 and SPS 40-10 absorb a greater amount of water and are therefore more porous. The lower water absorption and porosity of the MSS30-20 mortars are due to the better particle size distribution of the SS30-20 sand and the higher mass densities in the fresh state of these mixtures. Thus, these results show that better particle packing favors the compaction of the mortar in the fresh state, thus reducing the porosity in the hardened state. Comparing the results, the research by Aquino et al. [38] obtained water absorption values ranging from 15–15.5%, like the present research. Therefore, the results obtained are coherent.

#### 3.2.3. Weight Change Rate

**Figure 7:**
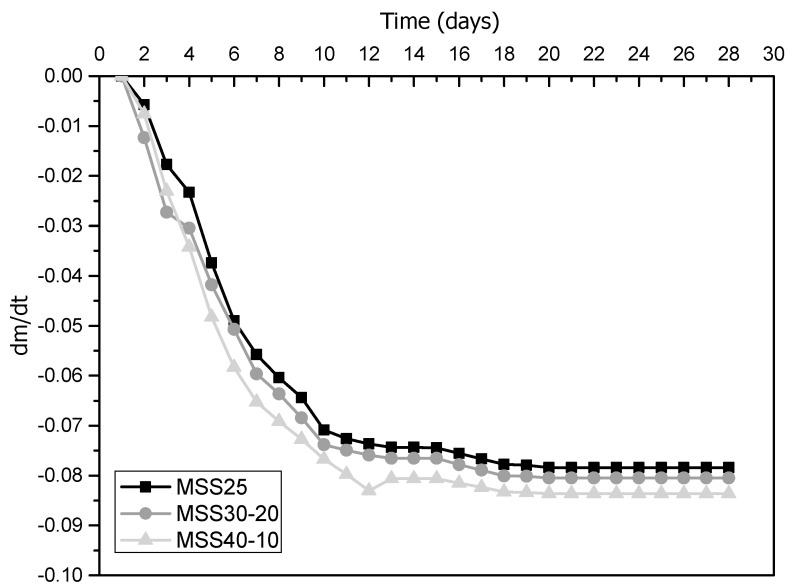
Weight change rate of hardened mortars.

The rate of weight variation (*dm*/*dt*) evaluates the behavior of the mortars regarding the loss and gain of weight during hardening. This behavior is related to the water retention capacity that, the higher it is, the lower the mass variation rate will be. The weight variation occurs due to a physical process in which the mixing water evaporates over time, being more evident in the initial stages of curing [16], as shown in Figure 7.

The behavior of the mortars studied is similar, especially those produced with sands with better particle size composition (SS25 and SS30-20), which is beneficial for the hardening of the cement and contributes to preventing the appearance of cracks by drying shrinkage. However, mortars with higher percentages of “coarse” particles (MSSP40-10) showed higher *dm*/*dt* throughout the test period, i.e., they have a lower capacity to retain water inside. The reason for this worse performance can be explained in two ways. First, the higher amount of water in these mixtures was a determining factor. Second, the SS40-10 sand has more voids between its particles (lower uniformity coefficient and bulk density) and the mortars are less dense when compared to the others. These results corroborate those of water absorption and porosity and confirm that these properties are related to the weight variation rate of mortars.

#### 3.2.4. Compressive Strength and Flexural Strength

**Figure 8:**
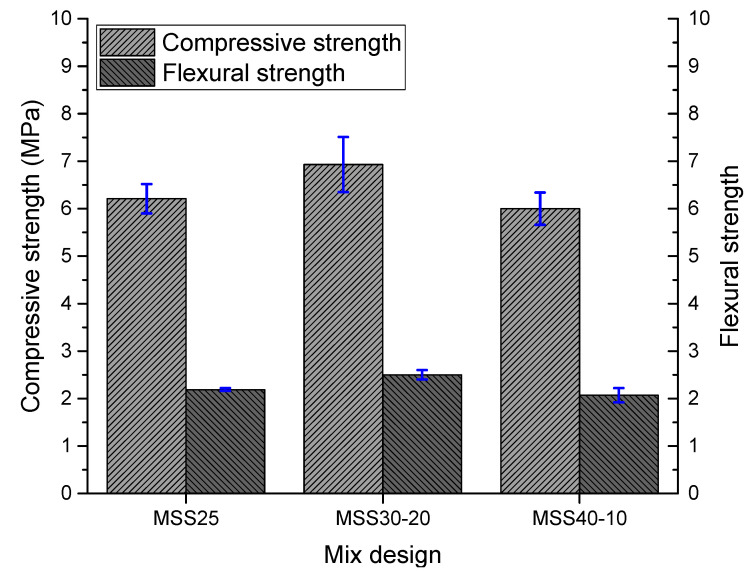
Results of compressive and flexural strength tests.

The results of the compressive strength (*f*_c_) and flexural strength (*f*_t_) of the mortars studied are shown in Figure 8. In terms of compressive strength classification established by NBR 13281 [54], the mortars studied fall into classes P4 (4.0 to 6.5 MPa) and P5 (5.5 to 9.0 MPa). Compared to the European standard (EN 998-1 [55]) that specifies requirements for mortars for internal and external rendering, the mortars studied have wider applications, since they belong to classes CS III and CS IV and, therefore, are suitable for most of the applications established by the aforementioned standard.

The mortars produced with SS40-10 have lower mechanical strengths compared to the other mortars. This shows that it is possible to increase the content of “coarse” and “fine” fractions in the standardized sand up to the limit of 30% and 20%, respectively. Higher levels, as is the case of mixture PS40-10 results in a worse arrangement of the granular skeleton and, consequently, contribute to obtaining mortars with higher water consumption, lower compaction in the fresh state, and lower mechanical strengths.

The better mechanical performance of MSS30-20 is due to the better behavior of SS30-20 sand in terms of particle size distribution, uniformity coefficient, and bulk density. All these parameters contribute to obtaining an aggregate fraction with a lower amount of voids and, therefore, mortars with lower water demand, with higher densities (more packed), less porous and with higher mechanical strengths. For Santos et al. [35] when using aggregates with better particle size distribution the best mechanical performance occurs through two mechanisms: (i) filling of voids, where the voids between medium-sized grains are filled by smaller grains and (ii) chemical reaction that improves the interfacial transition zone (ITZ).

Sugrañez et al. [22] reveal that the compressive strength decreased with increasing particle size, indicating that the microstructure was a consequence of a primitive arrangement in the raw materials. As a result, the mortars were more compact and therefore stronger. These authors also conclude that the smaller the average pore diameter, the higher the compressive strength, which justifies the results obtained in the immersion water absorption test.

It is noteworthy that the results obtained are like the research by Ferreira et al. [26,27], where the results for compressive strength were 5–6.5 MPa and the results for tensile strength in bending were 1.78–2.25 MPa, compatible with the results obtained in this research.

#### 3.2.5. Dynamic Modulus of Elasticity

**Figure 9:**
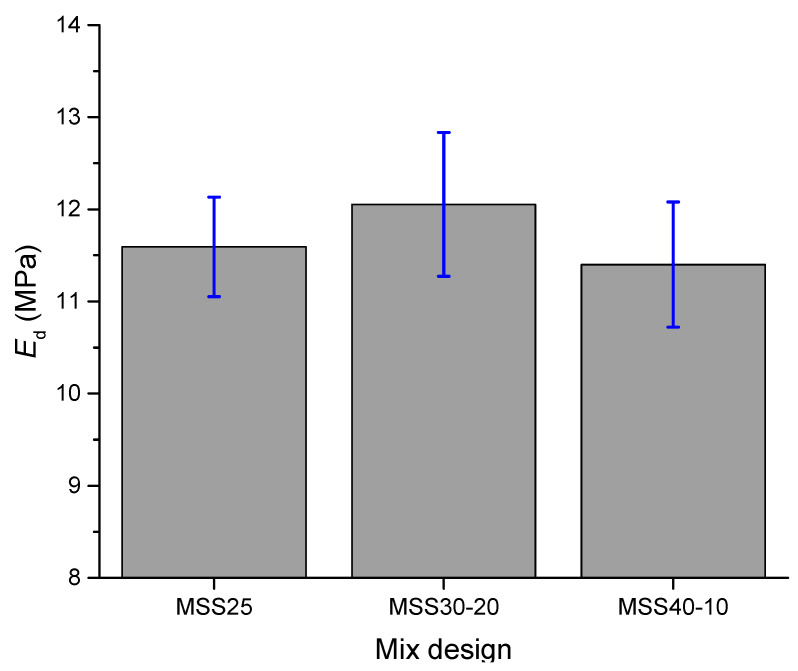
Results of mortar dynamic modulus of elasticity tests.

The dynamic modulus of elasticity (*E*_d_) of the mortars is shown in Figure 9. Note that the results obtained are similar due to the use of aggregates of the same mineralogical nature, which is directly related to the stiffness of the aggregates and, consequently, to the modulus of deformation of the mortars. However, the highest *E*_d_ was obtained by the mortars produced with MSS30-20 due to the increased stiffness of the internal structure of the mortar caused by the packing of the particles.

These results are consistent with the mechanical strength of mortars and demonstrate that stronger mortars are more rigid. From the point of view of durability of mortar coverings, this is not a desirable characteristic, because, as a rule, stiffer coverings have less capacity to absorb deformations and, therefore, are more prone to cracking, besides facilitating the access of water and aggressive agents. However, these mortars could be used as structural repair material.

On the other hand, the parameter proposed by the Centre Scientifique et Technique du Bâtiment (CSTB) can better characterize the behavior of mortars in terms of cracking. One of the criteria proposed by CSTB is based on the ratio between the modulus of elasticity and flexural strength (*E*/*f*_t_). It is known that a low modulus of elasticity contributes to better deformability, while a high flexural strength indicates better mechanical strength to support the applied load, therefore, the mortar tends to present a higher susceptibility to cracking when the ratio *E*/*f*_t_ is high [56]. The results shown in Table 5 indicate that although the MSS30-20 mortars can be considered stiff, the *E*/*f*_t_ ratio is lower when compared to the other mortars, which suggests a more ductile behavior and less susceptibility to cracking. This shows that the higher flexural strength may be important to reduce the susceptibility to cracking of mortars that have higher modulus of deformation.

#### 3.2.6. Performance Analysis

**Figure 10:**
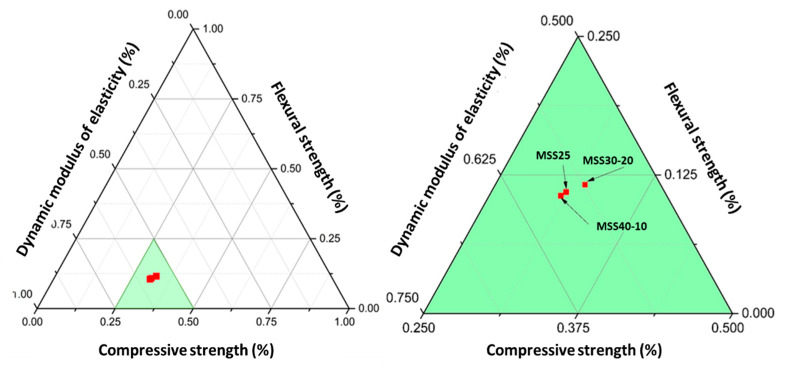
Ternary diagram of the results in the hardened state.

In order to determine the mix proportion with the better performance of the standardized mortars, a comparative analysis of the studied hardened properties was initially performed. For this analysis, the results of compressive strength (*f*_c_), flexural strength (*f*_t_), and dynamic elastic modulus (*E*_d_) tests of the samples at 28 days were selected. Then, the data obtained were normalized and presented by means of a ternary diagram, as shown in Figure 10. For data normalization, the method proposed by [57] was used, in which first the absolute sum of each value of the selected hardened properties is performed, and then the value of the contribution share of each property to a total value universe (1) is calculated. The normalization of the results is presented in Table 6.

The results shown in Figure 10 reveal that the mortar MSS30-20 presents better performance, i.e., it has higher values of ft and lower values of Ed, which is important to obtain mortars with greater ability to absorb deformations and, therefore, less susceptible to cracking. By the Absolute Sum analysis (Table 5) there was an improvement of 7.5% and 10.3% in the mortar MP30-20 in relation to the mortars MSS25 (REF) and MSS40-10, respectively.

These results confirm that the better packing of the SS30-20 sand particles contributes to obtaining mortars with better performance.

## 4. Conclusions

This work analyzed the effects of using standard sand defined by NBR 7214 [58] with different amounts of “coarse” and “fine” fractions on the physical and mechanical behavior of mortars. From the experimental results and discussions above, the following conclusions can be drawn:

- The bulk density and the uniformity coefficient are important parameters for predicting the physical packing of the sand particles and, consequently, the physical and mechanical behavior of the mortars;
- The variation in the particle size distribution of the standardized sands did not cause major changes in the bulk density of the mortars. However, the SS30-20 standardized sand (30% and 20% of “coarse” and “fine” fractions, respectively), obtained a better content of incorporated air of approximately 0.5%;
- In the hardened state, the SS30-20 sand obtained better performance in all analyzed properties. This behavior was due to the better particle size distribution of this sand compared to the other sands;
- Although the mortars with SS30-20 showed higher dynamic modulus of elasticity (*E*_d_), based on the *E*/*f*_t_ ratio these mortars have higher deformation capacity and therefore have lower susceptibility to cracking. The *E*/*f*_t_ ratio for the MSS30-20 composition was 4815, while for the MSS-25 composition, they were 5304, illustrating this information.

These results show that the physical and mechanical behavior of the mortars was improved when using 30% and 20% fractions of “coarse” and “fine” particles in the composition of standardized sands, respectively. Nevertheless, the discussions and conclusions obtained in this research reveal that the dosage of particle size fractions to obtain standardized sands according to the criteria established by NBR 7214 [58] does not cover all the available natural sand compositions.

Therefore, the dosage of the particle size fractions that constitute standardized sand should consider the optimization of the granular skeleton (greater packing of the grains), being the unit mass and the granulometric composition of the aggregates important parameters to guarantee this premise and to obtain economic mixtures and mortars with better physical and mechanical performances.

## Figures and Tables

**Table 1:** Particle size fractions and nomenclatures of standardized sands.

	% “Coarse” Fraction		% “Fine” Fraction	
Standardized sands	Sieve #16	Sieve #30	Sieve #50	Sieve #100
	(2.4–1.2 mm)	(1.2–0.6 mm)	(0.6–0.3 mm)	(0.3–0.15 mm)
SS-25 (REF)	25%	25%	25%	25%
SS30-20	30%	30%	20%	20%
SS40-10	40%	40%	10%	10%

**Table 3:** Mix design of the mortars.

Mix Designation	Composition (g)							
	Binder		Fine Aggregate				Water	w/b
	PC	HL	Sieve #16	Sieve #30	Sieve #50	Sieve #100		
MSS25	375	375	562.5	562.5	562.5	562.5	576	0.77
MSS30-20	375	375	675	675	450	450	564	0.75
MSS40-10	375	375	900	900	225	225	585	0.78

**Table 4:** Mix design of the mortars.

Properties	Standardization	No. of Samples	Specimens
Bulk density	NBR 13278 [40] ^1^	3	Fresh mortar
Air content	NBR 13278 [40] ^1^	3	Fresh mortar
Capillary water absorption	NBR 15259 [41] ^2^	3	Hardened mortar
Water absorption by immersion	NBR 9778 [42] ^3^	3	Hardened mortar
Weight change rate	CSTB [43]	3	Hardened mortar
Flexural strength	NBR 13279 [44] ^4^	3	Hardened mortar
Compressive strength	NBR 13279 [44] ^4^	6	Hardened mortar
Dynamic modulus of elasticity	NBR 15630 [45] ^5^	3	Hardened mortar

^1^ Equivalent to EN 1015–6 (1998); ^2^ Equivalent to EN 1015–18 (2002); ^3^ Equivalent to EN 1936 (2007) and ASTM C642 (2013); ^4^ Equivalent to EN 1015–11 (1999) and ASTM C1314 (2016); ^5^ Equivalent to ASTM C597 (2009) and EN 12504–4 (2004).

**Table 5:** Susceptibility of mortars to cracking.

Properties	*f*_t_ (MPa)	*E*_d_ (GPa)	*E*/*f*_t_
MSS25	2.19	11.593	5.304
MSS30-20	2.50	12.054	4.815
MSS40-10	2.07	11.401	5.503

**Table 6:** Normalized results of the hardened mortar properties for 28 days.

Mixes Proportions	*f*_c_ (MPa)	*f*_t_ (MPa)	*E*_d_ (GPa)	Absolute Sum
MSS25	0.3107	0.1096	0.5798	19.99
MSS30-20	0.3226	0.1163	0.5612	21.49
MSS40-10	0.3080	0.1063	0.5857	19.48

## Data Availability

Not applicable.
